# Peers empowering peers-feasibility of a peer educator training program to prevent diabetes

**DOI:** 10.1186/s12905-022-01645-w

**Published:** 2022-03-09

**Authors:** Eva Vivian, Constance Flanagan

**Affiliations:** 1 School of Pharmacy, University of Wisconsin-Madison, 777 Highland Avenue, Madison, WI 53705 USA; 2 School of Human Ecology, University of Wisconsin-Madison, Madison, WI 53706 USA

**Keywords:** Obesity, Prediabetes, Diabetes Prevention Program, Peer support, Grandmothers, Caregivers, Type 2 diabetes, Children

## Abstract

**Background:**

This study assessed the feasibility and acceptability of the Healthy Outcomes through Peer Educators (HOPE) training program designed to train African American (AA) grandmothers to serve as peer educators for other AA grandmothers who are at risk for diabetes. The newly trained peer educators will provide the needed encouragement and positive reinforcement essential for successful sustainable health behaviors to grandmothers enrolled in a subsequent Diabetes Prevention Program.

**Methods:**

Thirty AA women between the ages of 49–72 were enrolled in the pre-post single group study. The participants were required to attend a 2-h Diabetes Prevention Program session every week for 16 weeks. The sessions covered the role of the peer educator and encouraged participants to increase their physical activity and consume more servings of fruits and vegetables daily. We noted the number of participants who successfully completed the training and solicited their perspective regarding the strengths of the program and suggestions to improve the program. The Wilcoxon Signed Ranks Test was performed to evaluate changes in body weight, self-reported physical activity, and fruit and vegetable intake from baseline to week 16.

**Results:**

Ninety three percent (n = 28) of enrollees completed the training program. All (n = 28) the peer educator trainees indicated that they would recommend the program to a friend and 93% (n = 26) reported that the program met their needs. The participants (n = 28) felt that the training prepared them to offer support to other grandmothers in their community. A Wilcoxon Signed Ranks Test revealed that participants body weights were significantly lower after completion of the 16 week training program (median = 156.5 lbs., n = 28) compared to baseline (median = 163.0, n = 28), Z = − 4.43, *p* < 0.05. Fruit and vegetable intake increased significantly (median = 5, n = 28) at week 16 compared to week one, (median = 2, n = 28) Z = 4.46, *p* < 0.05. Participants were more physically active at week 16 (median = 150 min per week, n = 28) compared to week 1, (median = 30 min per week, n = 28) Z = 4.62, *p* < 0.05.

**Conclusions:**

The peer educator training component of HOPE was found to be feasible and acceptable as evidenced by our successful recruitment and retention of participants. Participation in the peer educator training program also resulted in improvement in health behaviors.

## Introduction

Almost one third of African American (AA) grandmothers living with grandchildren serve as primary caregivers compared to 9% of the general population [1]. African American grandmother caregivers represent a vulnerable population that is more likely to live in poverty when compared to women of other racial/ethnic groups across all age groups [2–4].

The incidence of obesity, depression, and diabetes is high among AA grandmother caregivers [4]. The psychological stress associated with raising a grandchild and maintaining a household may exacerbate pre-existing health conditions or induce unhealthy behaviors that worsen existing health conditions or increase the risk for additional health problems later in life [5–10].

It is likely that 80% of AA grandmother caregivers are overweight or obese, many whom care for a grandchild who is also overweight or obese [11, 12]. The risk of overweight or obese AA children remaining so in adulthood is at least twice that for normal weight children, increasing their risk for developing diabetes, heart disease, and other obesity related complications [12, 13]. Grandmothers influence their grandchildren’s eating patterns by modeling their own eating behaviors and food preferences [14–16]. Overweight or obese AA grandmothers who are caregivers for their grandchildren may benefit from interventions to reduce obesity and prevent diabetes, and improvements in their lifestyles may translate to benefits in reducing the alarming obesity trend among African American grandchildren, however this has not been tested [13, 14].

Most AA grandmothers serve as the primary caregiver for their grandchild, often receiving little or no support from other family members [17]. Therefore, a grandmother who becomes disabled due to poor health may be forced to place their grandchild in foster care (either temporarily or permanently) or leave the child with an unreliable parent or relative, compromising the well-being of the child and imposing financial costs on the public sector [17, 18]. There is an urgent need for preventive care interventions for these ‘hidden figures’ who provide household maintenance and childcare for their grandchildren.

The Diabetes Prevention Program (DPP) [19] is an evidence-based, lifestyle change program that has been shown to decrease the risk of diabetes, through weight loss among all participants, including African Americans. However, suboptimal weight loss outcomes have been reported among AAs, particularly older women who participated in DPP translations [20]. High attrition and low attendance may have contributed to these outcomes, indicating additional enhancements are needed to optimize outcomes for AAs, particularly AA grandmothers [20]. In a previous study, AA mothers and grandmothers who were the sole economic and parenting provider within the home recommended using peer support as a means to provide a positive sense of accountability and confidence to make lifestyle changes that result in weight loss [21]. Based on this feedback, we propose augmenting the DPP with a novel peer support program, Healthy Outcomes through Peer Educators (HOPE) where grandmother caregivers serve as peers to other grandmother caregivers, helping to build self-efficacy and providing the needed encouragement and positive reinforcement essential for successful sustainable health behaviors within a low resource environment. Several studies have reported that incorporating peer support into structured educational programs results in improved health behaviors and disease management.in adults [22–26]. HOPE provides a great framework for creating a healthy living intervention because grandmothers who live in the same community and share similar characteristics and experiences are well positioned to support each other in their efforts to lead a healthy lifestyle [25, 26]. Since AA grandmothers are the most revered members of the AA community who serve as a source of heritage and wisdom [1], empowering them to serve as positive role models should facilitate behavior change and healthy behaviors for themselves and their children [18].

Our peer support intervention is based on the Social Learning Theory (SLT) that assumes that people are shaped by their environment and learn by observing others [27, 28]. According to SLT, the behavior of a model must grab the attention of the participant before observational learning can occur. The participant must remember or retain the behavior before reproducing the model’s behavior. More importantly, the participant must be motivated by something or someone who has more value than the effort to reproduce the behavior.

**Fig. 1:**
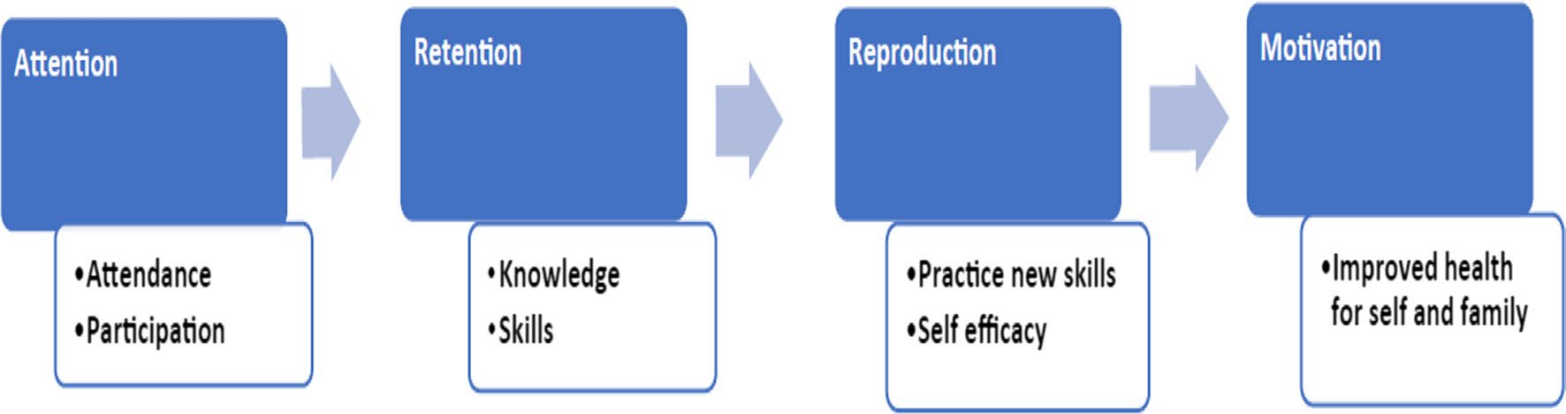
Steps of Social Learning Theory [27, 28]

Grandmothers enrolled in DPP will increase knowledge, practice new skills, and increase self-efficacy as a result of receiving ongoing peer support (Fig. 1). In a previous study, grandmothers stated that their greatest motivation to take steps to improve their health was their grandchild(ren) whom they loved dearly [29].

The first step toward the delivery of DPP + HOPE is to train AA grandmothers to serve as peer educators for other grandmothers within their community. This paper provides the outcomes of this study which assessed the feasibility of the novel peer educator training component of HOPE.

## Methods

### Sample

This pre-post single group study tested the feasibility and acceptability of the 16-week peer educator training component of the HOPE intervention. The study was approved by the University Social Sciences Human Subjects Protection Committee and participants provided written informed consent prior to any study procedure.

Any woman who self-identified as African American was eligible to participate in the training program if she was 40 years of age or older and identified as the primary caregiver of one or more grandchildren ages 2 through 18 years. A primary caregiver was defined as “one who provides instrumental and expressive care to a grandchild living in the same household daily for an indefinite period.” [29] In addition, women interested in becoming a peer educator had to meet eligibility requirements for participating in DPP which included: (1) being overweight or obese (BMI ≥ 25 kg/m^2^); (2) no previous diagnosis of diabetes; 3) and a glycosylated hemoglobin A1C between 5.7 and 6.4% [19]. The grandmother must be willing to complete a demographic questionnaire and two validated surveys that assess physical activity and dietary behavior.

The grandmother must be willing to be weighed at the time of enrollment in the study and the end of the study period as well as attend weekly DPP training sessions as able. Grandmothers were excluded if they were pregnant or had diseases that would limit their life span or restrict their ability to participate in the study.

We recruited 30 women from two community centers located in ethnically diverse neighborhoods in Wisconsin. The staff at each community center was asked to identify 15 women whom they felt would be a good peer educator for the HOPE peer educator training. In addition to meeting the inclusion criteria listed above for grandmothers, desirable characteristics and attributes of a peer educator included being actively engaged in community-based activities, being willing and available to be trained and participate in the subsequent DPP + HOPE intervention, have good interpersonal skills, possess similarities to the target participants, be respected in the community, have the ability to motivate others, have good listening skills, have basic problem-solving skills, live in the community being served, and be willing to help the community.

### Peer support training

The grandmothers were required to participate in a 32-h DPP training program. The training program’s schedule is flexible. The training program can be offered in its entirety on 4 consecutive days for trainees who prefer a concentrated experience or spread out over a 2-to-16-week period. The HOPE training program was offered 2 h a week over 16 weeks to accommodate the participants schedules.

The sessions which were led by a diabetes care and education specialist, promoted healthy eating, increased physical activity, modest weight loss (5%), and reduction in hemoglobin A1C. The sessions also covered the role of the peer educator which included empathic listening and helping participants develop health goals for themselves and family members. These training sessions were guided by the DPP training manual and a peer support training manual and toolkit that focused on supportive, non-judgmental communication, goal setting, motivational interviewing, and providing social and emotional support. The grandmothers were also required to complete human participant research ethics (IRB) training.

### Measures

We assessed the feasibility of the training program by noting the number of participants recruited and retained in the program. We also conducted open-ended interviews during the last week of the program to learn about each participant’s experience during the training sessions. The 30-min interview was led by a study team member who did not participate in the training sessions to encourage honest feedback. Based on our previous work [21], we asked participants to share their views regarding: (a) beneficial or useful aspects of the program; (b) problems or difficulties experienced during the program; (c) recommendations to improve the program; and (d) if they would recommend the program to a friend. The interviews were audio recorded and transcribed. Participants who dropped out of the program were interviewed by phone to learn (a) why they left the program, (b) aspect of the program they liked or disliked, and (c) recommendations for improving the program [21].

A digital electronic scale (Conair Body Analysis Weight Tracker Scale Model CON WW89T) was used to measure the body weight of each participant. Each grandmother’s weight was obtained while the woman was in a standing position, shoeless, and wearing light clothing. Physical activity and dietary behavior were assessed at the time of enrollment and the completion of the 16 week training period using two validated surveys: the International Physical Activity Questionnaire (IPAQ) [30] and the Food Frequency Questionnaire [31]. The IPAQ estimates levels and frequency of physical activity during the past 7 days, and the Food Frequency Questionnaire contains questions about customary intake of various food groups [30, 31].

Hemoglobin A1c was evaluated at baseline to determine eligibility to participate in the study [19] and the end of the study to determine if the participant progressed to diabetes. We used the A1cNow + system, the National Glycohemoglobin Standardization Program Certified, CLIA-waived, system that provides results using a finger stick test.

### Analysis

The authors read the transcripts from each interview thoroughly to obtain an understanding of the data. Analysis of the interview transcripts used a conventional content analysis approach [32], where each transcript was coded by hand with notations to delineate a basic description for each idea. The principal investigator (first author) and the co-investigator (second author) coded independently, then met to discuss their impressions and reach consensus about the overall meaning of the content. Finally, the authors identified common themes that emerged from the content analysis.

Because the data was slightly skewed, the Wilcoxon Sign Rank Test was used to assess changes in body weight, physical activity and fruit and vegetable intake from baseline to week 16. All analyses were conducted using SAS (SAS Institute Inc, Cary NC).

## Results

Participants were, on average, 52.4 ± 9.5 years old (49–72 years) and all reported annual family incomes less than 200% of the 2019 federal poverty level. The participants reported having a total of 42 grandchildren (1 infant, 38 school age children, and 3 unspecified age) living with them. Of these children, 60% were boys.

### Adherence and participation

Thirty women were recruited to participate in the training program. Ninety-three percent (n = 28) successfully completed the training. One woman relocated to another state during week 3 and another left the program during week 2 due to an employment opportunity.

The average weekly participation rate varied between 96 and 82% which leveled off at after week four of the study.

### Evaluation of the program

All study participants (n = 28) were available to evaluate the program at the end of the 16 week study period. Participants unanimously agreed that they would recommend the program to a friend and most (94%, n = 26) reported that the program met their needs. All the participants reported that they really enjoyed interacting with their neighbors during the peer educator training sessions and felt that the training prepared them to offer support to other grandmothers in their community.

A significant theme that emerged from the exit interviews was that participants enjoyed the training sessions because they were able to connect with neighbors and offer support, strength, and hope to their peers, which allowed for personal growth, wellness, and a sense of belonging within the community. One peer educator trainee stated the following “When I moved to Wisconsin, I felt lonely because I didn’t know anybody. I watched a kid being picked on by other boys, but I did not know who to call for help. I knew a lot of my neighbors in Chicago, Illinois so I could call them if I saw their kid in trouble.” Several grandmothers suggested that we include the children in some of the sessions, particularly the cooking and nutrition classes. One grandmother stated “I want to learn how to cook green vegetables like asparagus and Brussels sprouts. I think I overcook vegetables and I add too much salt to give them taste. I know they are full of vitamins but they cost so much and don’t really fill you up.” 

A Wilcoxon Signed Ranks Test revealed that participants body weights were significantly lower after completion of the 16 week training program (median = 156.5 lbs., n = 28) compared to baseline (median = 163.0, n = 28), Z = − 4.43, *p* < 0.05. Fruit and vegetable intake increased significantly (median = 5, n = 28) at week 16 compared to week one, (median = 2, n = 28) Z = 4.46, *p* < 0.05. Participants were more physically active at week 16 (median = 150 min per week, n = 28) compared to week 1, (median = 30 min per week, n = 28) Z = 4.62, *p* < 0.05 (Table 1).

**Table 1:** Results-changes in body weight and health behaviors

	Pre (week 1) (baseline)	Post (week 16)	*p* value
Age (years)	52.4 ± 9.5		
Median body weight (lbs)	163 lbs	156.5 lbs	< 0.05
Median physical activity (minutes per week)	30	150	< 0.05
Median fruit and vegetable intake (servings per day)	2	5	< 0.05

In addition to positive measured health outcomes, participants reported an increase in family time through walks, active play, reading with their grandchildren and preparing and eating meals as a family. Participants frequently mentioned that their grandchild became a ‘change agent’ by refocusing them on healthy behaviors and practices. At one site, the peer educator trainees led a petition to remove the soda vending machines from the local community center, improving their built environment and ultimately advancing community health.

## Discussion

This article describes a peer educator training program designed to provide grandmothers with the skills to help other grandmothers in their community in making and sustaining healthy lifestyle choices. These newly trained peer educators will be paired with peers enrolled in a subsequent structured educational intervention, DPP + HOPE to improve problematic health behaviors related to diet and exercise in grandmother caregivers. These peer educators will lead group activities during the DPP sessions and support their peers in making and sustaining healthy lifestyle choices. Peer educators will also provide one on one phone support during the week to their assigned peers to reinforce DPP session topics and encourage participants to maintain health goals. During these phone calls, participants can discuss any barriers they face when attempting to meet their goals, and collectively problem solve ways of addressing the barriers. All the participants expressed confidence and enthusiasm about providing support to other grandmother caregivers in their community.

The peer educator training component of HOPE was found to be feasible and acceptable as evidenced by our successful recruitment and retention of 93% of participants (n = 28) who successfully completed the study. The peer educator trainees enjoyed interacting with their neighbors and reported increasing their physical activity and consumption of fruits and vegetables because of their participation in the program. Weight gain among the participants was attenuated during their enrollment in the training program with 89% (n = 25) of participants experiencing weight loss (Table 1).

Peer support has been found to improve health behaviors and disease management when incorporated into an educational program [22–26]. Our results are similar to a feasibility and acceptability study titled “Supportive Text-Messaging Against Regain (STAR)” project [33]. STAR was a 16 week, community-based, combined peer group and text message–based intervention study that utilized mobile technology and in-person group meetings to deliver health information, encouragement, and support to achieve goals of weight loss and management among 22 African American women. The study reported modest, but significant reductions and body weight and body mass index [33].

There are several limitations to this study. First, the study findings cannot be generalized to all AA women because of the small sample size, sample selection, and low statistical power. However, this small sample is acceptable since this was a feasibility study. A larger study is needed to more rigorously determine intervention efficacy and the generalizability of the results; We plan to conduct a randomized controlled trial comparing the intervention efficacy of DPP + HOPE with an active comparator, DPP alone.

It is important to note that self-report of physical activity and fruit and vegetable intake possibly introduced social desirability and recall bias and compromised the validity of the findings. For future studies, a research associate who does not interact with the participants will administer the survey to each participant individually, so participants are not pressured to impress the research staff or peers. We will ask participants to maintain food diaries, so they have a record of their food intake. Fitbit watches will be provided for all participants (peer educators and participants) so a record of steps walked per day is maintained.

The peer coach training for HOPE was approximately 40 h in total. The community advisory committee and study participants from a previous study recommended that the peer training be limited to 2 h per week to give the participants adequate time to learn the material. The training program can be translated to other communities and the trainings sessions can be shortened or lengthened based on the educational background and availability of the participants.

The strengths of this study which include our intent to assess the feasibility of recruiting and retaining high-risk grandmother caregivers in the training program outweigh the limitations noted above.

The HOPE training program was designed to be sustainable at a community or neighborhood level by being responsive to each community’s unique physical and social environment. Peers can be matched based on culture, language, age, and sex. Churches, civic groups, and community centers can partner with health care organizations and academic partners to train neighborhood volunteers with a DPP based curriculum at little to no cost. This project promoted strong social and community ties among community members strengthening social capital and providing participants with the confidence to advocate for healthier food choices at their local community center. The success of the HOPE training and the community’s excitement to enlarge the project by augmenting an existing DPP program with HOPE reinforces its potential for other communities.

## Conclusion

Incorporating community-based peer support in educational programs may promote strong social and community ties among community members. By sharing their own lived experiences, peer educator grandmothers can help other caregivers in their community develop their own health goals, create strategies for self-empowerment and take steps toward serving as positive role models for their grandchildren. This work may provide a model for effectively facilitating behavior change and reducing the risk of obesity, diabetes, and other chronic diseases.

## Data Availability

The datasets analyzed during the current study available from the corresponding author on reasonable request.

## Abbreviations

AA: African American
HOPE: Healthy Outcomes through Peer Educators
DPP: Diabetes Prevention Program

## References

[1] 2018 AARP. Grandparents Today National Survey. 2019. https://www.aarp.org/content/dam/aarp/research/surveys_statistics/life-leisure/2019/aarp-grandparenting-study-african-american-black.doi.10.26419-2Fres.00289.003.pdf. Accessed 13 Aug 2020.

[2] Baker LA, Silverstein M, Putney NM (2008). Grandparents raising grandchildren in the United States: changing family forms, stagnant social policies. J Soc Policy.

[3] Pandey A, Littlewood K, Cooper L, McCrae J, Rosenthal M, Day A (2019). Connecting older grandmothers raising grandchildren with community resources improves family resiliency, social support, and caregiver self-efficacy. J Women Aging.

[4] Minkler M, Fuller-Thomson E (2005). African American grandparents raising grandchildren: a national study using the Census 2000 American Community Survey. J Gerontol B Psychol Sci Soc Sci.

[5] Prokos AH, Keene JR (2012). The life course and cumulative disadvantage: poverty among grandmother-headed families. Res Aging.

[6] Lipscomb RC (2005). The challenges of African American grandparents raising their grandchildren. Race Gender Class.

[7] Kelley SJ, Whitley DM, Campos PE (2013). African American caregiving grandmothers: results of an intervention to improve health indicators and health promotion behaviors. J Fam Nurs.

[8] Hayslip B, Kaminski PL (2005). Grandparents raising their grandchildren: a review of the literature and suggestions for practice. Gerontologist.

[9] Baker LA, Silverstein M (2008). Depressive symptoms among grandparents raising grandchildren: the impact of participation in multiple roles. J Intergener Relatsh.

[10] Musil C, Warner C, Zauszniewski J, Wykle M, Standing T (2009). Grandmother caregiving, family stress and strain, and depressive symptoms. West J Nurs Res.

[11] Hales CM, Carroll MD, Fryar CD, Ogden CL (2017). Prevalence of obesity among adults and youth: United States, 2015–2016. NCHS data brief, no 288.

[12] Singh AS, Mulder C, Twisk JW, Mechelen W, Chinapaw MJ (2008). Tracking of childhood overweight into adulthood: a systematic review of the literature. Obes Rev.

[13] Whitaker RC, Wright JA, Pepe MS, Seidel KD, Dietz WH (1997). Predicting obesity in young adulthood from childhood and parental obesity. N Engl J Med.

[14] Dhana K, Haines J, Liu G (2018). Association between maternal adherence to healthy lifestyle practices and risk of obesity in offspring: results from two prospective cohort studies of mother–child pairs in the United States. BMJ.

[15] Pulgaron ER, Marchante AN, Agosto Y, Lebron CN, Delamater AM (2016). Grandparent involvement and children's health outcomes: the current state of the literature. Fam Syst Health.

[16] Thomaseo BE, Wilder T, Beech BM, Bruce MA (2017). Caregiver feeding practices and weight status among African American adolescents: the Jackson Heart KIDS Pilot Study. Eat Behav.

[17] Parks EP, Kazak A, Kumanyika S, Lewis L, Barg FK (2016). Perspectives on stress, parenting, and children's obesity-related behaviors in Black families. Health Educ Behav.

[18] Kelley SJ, Whitley DM, Campos PE (2013). Psychological distress in African American grandmothers raising grandchildren: the contribution of child behavior problems, physical health, and family resources. Res I Nurs Health.

[19] The Diabetes Prevention Program Research Group (2002). Reduction in the incidence of type 2 diabetes with lifestyle intervention or metformin. N Engl J Med.

[20] Samuel-Hodge CD, Johnson CM, Braxton DF, Lackey M (2014). African Americans and DPP translations. Obes Rev.

[21] Vivian EM, Colbert L, Remington PL (2013). Lessons learned from a community based lifestyle intervention for youth at risk for diabetes. J Obes Weight Loss Ther.

[22] Heisler M (2007). Overview of peer support models to improve diabetes self-management and outcomes. Diabetes Spectr.

[23] Wrobleski MM, Parker EA, Hager E, Hurley KM, Oberlander S, Merry BC, Black MM (2017). A community-driven implementation of the body and soul program in churches in the Twin Cities, Minnesota, 2011–2014. Prev Chronic Dis.

[24] Williams EM, Egede L, Oates JC, Dismuke CL, Ramakrishnan V, Faith TD, Johnson H, Rose J (2019). Peer approaches to self-management (PALS): comparing a peer mentoring approach for disease self-management in African American women with lupus with a social support control: study protocol for a randomized controlled trial. Trials.

[25] Schwartz CE, Sendor M (1999). Helping others helps oneself: response shift effects in peer support. Soc Sci Med.

[26] Fisher EB, Ayala GX, Ibarra L (2015). Contributions of peer support to health, health care, and prevention: papers from peers for progress. Ann Fam Med.

[27] Bandura A (1977). Social learning theory.

[28] Rosenstock IM, Strecher VJ, Becker M (1988). Social learning theory and the health belief model. Health Educ Q.

[29] Sands RG, Goldberg-Glen R, Thornton PL (2005). Factors associated with the positive well-being of grandparents caring for their grandchildren. J Gerontol Soc Work.

[30] The IPAQ Group. International Physical Activity Questionnaire. https://sites.google.com/site/theipaq/home. Accessed 13 June 2021.

[31] NutritionQuest. Block Food Frequency Questionnaire. https://nutritionquest.com/assessment/list-of-questionnaires-and-screeners/. Accessed 13 June 2021.

[32] Hsieh HF, Shannon SE (2005). Three approaches to qualitative content analysis. Qual Health Res.

[33] Lee S, Schorr E, Chi CL, Treat-Jacobson D, Mathiason MA, Lindquist R (2018). Peer group and text message-based weight-loss and management intervention for African American Women. West J Nurs Res.

